# Is Perceived Discrimination in Pregnancy Prospectively Linked to Postpartum Depression? Exploring the Role of Education

**DOI:** 10.1007/s10995-016-2259-7

**Published:** 2017-01-23

**Authors:** Irena Stepanikova, Lubomir Kukla

**Affiliations:** 1 Sociology Department, University of Alabama, Birmingham, USA; 2 Research Centre for Toxic Compounds in the Environment (RECETOX), Masaryk University, Brno, Czech Republic; 3 Institute for Research in Social Sciences, Stanford University, Stanford, USA

**Keywords:** Perceived discrimination, Postpartum depression, Socio-economic status, Education

## Abstract

*Objectives* The role of perceived discrimination in postpartum depression is largely unknown. We investigate whether perceived discrimination reported in pregnancy contributes to postpartum depression, and whether its impact varies by education level. *Methods* Prospective data are a part of European Longitudinal Study of Pregnancy and Childhood, the Czech Republic. Surveys were collected in mid-pregnancy and at 6 months after delivery. Depression was measured using Edinburgh Postnatal Depression Scale. Generalized linear models were estimated to test the effects of perceived discrimination on postpartum depression. *Results* Multivariate models revealed that among women with low education, discrimination in pregnancy was prospectively associated with 2.43 times higher odds of postpartum depression (*p* < .01), after adjusting for antenatal depression, history of earlier depression, and socio-demographic background. In contrast, perceived discrimination was not linked to postpartum depression among women with high education. *Conclusions* Perceived discrimination is a risk factor for postpartum depression among women with low education. Screening for discrimination and socio-economic disadvantage during pregnancy could benefit women who are at risk for mental health problems.

## Significance

*What is already known on this subject?* Perceived discrimination in pregnancy is linked to concurrent depression in cross-sectional studies but its predictive value for postpartum depression is largely unknown.

*What this study adds?* Using prospective data from a cohort of Czech pregnant women, we find that women with low education who report discrimination during pregnancy have 2.43 times higher odds of postpartum depression compared to their counterparts who do not report discrimination, even after controlling for antenatal depression, history of earlier depression, and socio-demographic background. These results suggest that perceived discrimination is an independent risk factor for postpartum depression among socio-economically disadvantaged women.

## Introduction

Postpartum depression is a common complication of childbearing (Robertson et al. 2003; Warner et al. 1996). Similar to depression during other life stages, depression during the postpartum period is characterized by persistent sadness, ahedonia, tearfullness, low energy, changes in eating and sleeping habits, poor concentration and memory, irritability, and suicidal ideation (Di Florio and Meltzer-brody 2015; Nonacs and Cohen 1998; Postpartum Depression: Action Towards Causes and Treatment (PACT) Consortium 2015). Women experiencing postpartum depression tend to feel overwhelmed by their parental role (Edhborg et al. 2005) and may have thoughts of harming their infant (Jennings et al. 1999). Suicides and homicides are rare among depressed women (Samandari et al. 2011); yet, suicide remains a leading cause of maternal death in high-income countries (Austin et al. 2007; Esscher 2013). Depressed women are less likely to breastfeed (Dennis & McQueen, 2007), use preventive pediatric care (Field 2010), and follow recommended safety practices, including using a car seat and covering electric outlets (Balbierz et al. 2015; McLearn et al. 2006). Moreover, they interact with their infants in ways that may adversely affect the motoric, cognitive, emotional, and social aspects of the child development (Beck 1998; Murray et al. 1996).

The etiology of postpartum depression is unclear (Meltzer-Brody 2011) but several risk factors have been identified. They include pre-pregnancy depression, depression and anxiety during pregnancy, stressful life events during the perinatal period, low social support, childcare stress, difficult infant temperament, low self-esteem, neuroticism, poor relationship with a partner, medical complications during pregnancy and delivery, and low socio-economic status (Robertson et al. 2003). Many of these factors are directly related to the stress experienced by new mothers, further emphasizing the significance of stress in postpartum depression.

Perceived discrimination is one type of stressor that has received growing scholarly attention in recent years. In studies with populations other than pregnant women, perceived discrimination was prospectively linked to depression and anxiety, as well as poorer health more generally (Paradies 2006; Pascoe and Richman 2009; Williams et al. 2003). For pregnant women more specifically, several cross-sectional studies revealed linkages between depressive symptoms and different types of perceived discrimination, including racial discrimination among Black pregnant women (Ertel et al. 2012), gender and economic discrimination among African–American and White women (Canady et al. 2008), and everyday discrimination due to any cause among low-income, low-medical risk Hispanics in Texas (Walker et al. 2012) and among low-income, inner-city women in Philadelphia (Bennett et al. 2010).

Yet, it remains unknown whether perceived discrimination in pregnancy prospectively predicts depression in later life. This is an important question since prior cross-sectional research cannot adjudicate causality. The observed associations could be present because perceived discrimination is a causal factors in later depression, because depression is a causal factor in the respondents’ tendency to report discrimination (for instance, because of recall bias), or because a third, unspecified, cause affects both depression and discrimination.

The goal of this study is to use longitudinal evidence to investigate the prospective relationships between perceived discrimination assessed in pregnancy and depression after delivery. Given the limited understanding of the role of perceived discrimination in depression, it is important to ground hypotheses concerning their relationship in an established theoretical framework. We turn to bio-psycho-social model specified by Clark et al. (1999); also see Brondolo et al. (2003) and Williams et al. (2003). The model argues that perceived discrimination contributes to negative health outcomes (not limited to depression), and that it does so via three mechanisms. First, perceived discrimination activates physiological stress responses. If overused, these responses may eventually lead to dysregulation of multiple bodily systems including those governing mental health. Second, perceived discrimination triggers negative emotions, such as anger, sadness, and guilt, contributing to poorer overall mental wellness. Third, victims of discrimination often face barriers to health care (Gee et al. 2009; Paradies 2006). They underutilize health services (Burgess et al. 2008), postpone medical tests and treatments (Van Houtven et al. 2005), forego preventive screenings (Gonzales et al. 2013; Trivedi and Ayanian 2006), and fail to adhere to medical recommendations (Haywood et al. 2014).

Consistent with the bio-psycho-social model, several studies report that discrimination during pregnancy predicts adverse health outcomes for the mother and inflant, such as preterm birth (Dole et al. 2003; Mustillo et al. 2004), low or very low birth weight (Collins et al. 1998, 2000; Dominguez et al. 2008; Mustillo et al. 2004), and increased stress reactivity of the infant (Thayer and Kuzawa 2015). We argue that all three mechanisms specified by the bio-psycho-social model are relevant also for postpartum depression. Stress and negative emotions are well-known correlates of depression (Bromberger and Matthews 1996; Feldman et al. 2009). Barriers to health care may prevent a postpartum woman with depressive symptoms from receiving proper medical evaluation and treatment. In sum, the application of the model to postpartum depression yields the following hypothesis: *Perceived discrimination during pregnancy is related to a higher likelihood of postpartum depression*.

Another focus of this study is on socio-economic status (SES) as a potential moderator of the relationship between perceived discrimination and depression. SES is a robust predictor of mental health. Lower education, a key indicator of lower socio-economic status, increases the risk of depression (Miech and Shanahan 2000) and higher rates of depression are evident among socio-economically disadvantaged pregnant and postpartum women more specifically (Goyal et al. 2010). Moreover, socio-economically disadvantaged individuals face an increased risk of discrimination (Watson et al. 2002; Ro and Choi 2009). These patterns are further complicated by race and lifestyle behaviors. Cheng, Cohen, and Goodman (2015), for instance, observed that White young adults with higher SES measured by parental education had lower rates of depression. Among their Black counterparts, however, the protective effects of socio-economic advantage were overwhelmed by the adverse effects of higher perceived discrimination. In another study, the impact of stress on mortality increased with smoking and physical inactivity among low SES individuals, but the same was not evident among high SES individuals (Krueger and Chang 2008).

Given the evidence on these complex inter-relationships, it is important to consider SES when evaluating linkages between depression and discrimination. Consistent with prior evidence on adverse mental health outcomes among low SES individuals, we hypothesize that *perceived discrimination has a stronger impact on postpartum depression among women with low education, compared to their highly educated counterparts*.

In evaluating these hypotheses, we account for the history of depression during pregnancy and during earlier life. This is important given the known role of earlier depression history in postpartum depression (Beck 2001; Gaillard et al. 2014; Robertson et al. 2004) and the evidence of cross-sectional linkages between perceived discrimination and depressive symptoms (Bennett et al. 2010; Canady et al. 2008; Ertel et al. 2012; Walker et al. 2012). Our approach enables us to understand whether perceived discrimination in pregnancy influences postpartum depression independently of depression history.

## Data

Data are a part of European Longitudinal Study of Pregnancy and Childhood, the Czech Republic (ELSPAC-CZ), a “longitudinal birth cohort study assessing children’s health from the foetal period until the age of 19” (Piler et al. 2016). ELSPAC was initiated by the World Health Organization with the goal to identify social, demographic, and environmental factors that affect health and well-being of children and their families in several European countries. It was conducted in accord with prevailing ethical principles and reviewed by ethical committees of participating institutions. The Czech sample consisted of women expected to give birth between March 1, 1991, and June 30, 1992 who resided in Brno and Znojmo regions of the Czech Republic as indicated by postal codes. Women were recruited by their gynecologists during a prenatal visit. At Time 1 (T1), baseline paper-and-pencil questionnaires were completed at around the 20th week of pregnancy by 4811 consenting participants. The questionnaire included measures of demographic background, depressive symptoms, self-reported history of major depression and other health-related measures. 3701 women remained in the study at Time 2 (T2), 6 months after delivery. After list-wise deletion of missing cases, the final analytical sample consisted of 3005 women.

To understand whether participants at 6 months differed from those who left the study after the baseline interview, we performed bivariate tests to assess whether attrition varied according to demographic and health-related characteristic. Compared to women who left the study, women who stayed in the study were older, more educated, lived in smaller households, were more commonly married and of European ethnicity. They were less likely to report discrimination and depression during pregnancy. They did not differ in regard to earlier history of depression, stress events, social support, and parity.

## Measures

Postpartum depression was measured at 6 months after delivery (T2) using Edinburg Postnatal Depression Scale (EPDS), a valid instrument commonly used to assess depression during pregnancy and the first year after delivery (Cox et al. 1987). In EPDS, respondents rate how they felt in the previous seven days using 10 items such as “I have felt sad or miserable” and “I have felt so unhappy that I have been crying” with a rating scale ranging from 0 (“never”) to 3 (“most of the time”). Following prior research (Affonso et al. 2000), the score of 13 or more on the additive index was taken to indicate probable depression (henceforth depression).

Perceived discrimination, the main explanatory variable, was measured in mid-pregnancy (T1). Respondents were asked “Would you say that during the past 12 months, someone treated you unfairly because of your gender, skin color, the way you dress, your family origin, speech, religious beliefs, or something else?” (yes/no). This measure captures the respondent’s perceptions of whether she experienced any discrimination (vs. no discrimination), regardless of the specific cause for discrimination, and is consistent with the literature suggesting that various types of discrimination are similar in that they are stressful and harm health (Chae et al. 2010; Krieger 1990, 1999).

Education serves as the key indicator of SES in this study. It is categorized as low (grade school or vocational training) versus high (secondary school or higher). The remaining variables were selected based on prior evidence concerning factors that contribute to depression. They were measured at baseline, following O’Hara and Swain (1996) and Robertson et al. (2003), who argue that the antenatal measurement of factors contributing to postpartum depression reduces bias. History of major depression was assessed by a question: “Have you ever had major depression? (yes/no).” Antenatal depression was measured in mid-pregnancy using EPDS. Models also adjusted for socio-demographic background, including age, ethnicity (European vs. non-European), household size, marital status (married vs. not married), and having children as a proxy for parity. Stress was evaluated using a list of 40 types of life events, e.g., death of a partner, conflict with law, divorce, and job loss. Pregnant women reported which events they experienced during the previous 9 months. The total of reported events was calculated. Finally, perceived social support was included because it buffers stress during pregnancy (Gosh et al. 2010). It was measured with 10 items designed specifically for ELSPAC and based on work by Thorpe, Dragonas, and Golding (1992) and Dragonas, Thorpe, and Golding (1992). Examples of items include “My partner provides the emotional support I need,” and “If I was in financial difficulty I know my family would help if they could.” Each item was rated on a 1–4 scale with response options “never feel,” “sometimes feel,” “often feel,” and “exactly feel.” The mean score was used to indicate the overall level of social support.

### Analytic Strategy

After obtaining descriptive and bivariate statistics, series of nested generalized linear models (GLM) with logit link function assessed whether perceived discrimination in pregnancy predicted postpartum depression. First, models for the whole sample were estimated. In Model 1, postpartum depression was modeled as a function of perceived discrimination, social support, prenatal stress events, and socio-demographic background. Model 2 added antenatal depression and earlier depression history to evaluate whether perceived discrimination predicted postpartum depression independently of depression before the delivery. Model 3 further added an interaction between perceived discrimination and education to test whether the effect of perceived discrimination varied by education levels. Finally, separate models for women with low versus high education were estimated to explore the differences in factors influencing postpartum depression in the two education groups. All analyses were conducted using Stata 14 statistical software.

## Results

Postpartum depression was observed in close to 5% of participants (Table 1 **)**. Women with postpartum depression were considerably more likely to report perceived discrimination, as well as antenatal depression and depression during their earlier life. They were more commonly of non-European ethnicity and nulliparous at baseline. They reported more life events and less social support.

**Table 1:** Characteristics of the sample

	No postpartum depression N = 2858 4.9%		Postpartum depression N = 147 95.1%	
	(%)		(%)	
Perceived discrimination	15.3		27.2***	
Low education	40.2		37.1	
European ethnicity	99.5		98.0*	
Has children	48.3		58.5*	
Married	88.6		90.5	
Antenatal depression	7.4		25.2***	
History of depression	4.9		11.6***	

	Mean	SD	Mean	SD
Age at delivery, years (15–49)	25.62	4.76	25.97	4.53
Household size (1–14)	3.25	1.29	3.53	1.61
Life events, prior 12 months (0–40)	3.11	3.75	4.03	3.72‡‡‡
Social support (1–4)	2.72	0.46	2.50	0.51‡‡‡

Data source: ELSPAC-CZ.* SD*  standard deviation. Ranges for continuous variables in parantheses

Pearson chi-square test comparing women with and without postpartum depression: *p < .05 , ***p < .001 (two-tailed)

Two-sample t-test comparing women with and without postpartum depression: ^‡‡‡^p < .001 (two-tailed)

Consistent with the first hypothesis, multivariate Model 1 reported in Table 2 reveals that perceived discrimination is a significant predictor of postpartum depression. The odds of postpartum depression increase by 71% (*p* < .01) for women who report discrimination compared to their counterparts who are similar on socio-demographic characteristics but do not report discrimination. After adjusting for depression during pregnancy and in earlier life in Model 2, a trend toward a positive relationships between perceived discrimination and postpartum depression remains evident at a marginal level of significance (*p* = .065).

**Table 2:** Estimates from general linear models of postpartum depression, whole sample

	Model 1			Model 2		
	OR	95% CI		OR	95% CI	
Perceived discrimination	1.71**	1.15	2.53	1.47	0.98	2.20
Low education	1.04	0.72	1.50	0.99	0.68	1.43
Age at delivery, years	1.00	0.96	1.04	0.99	0.95	1.04
European ethnicity	0.33	0.09	1.24	0.35	0.09	1.38
Has children	1.21	0.81	1.81	1.22	0.81	1.84
Married	1.54	0.85	2.81	1.78	0.97	3.27
Household size	1.12	0.99	1.26	1.12	0.99	1.26
Life events, prior 12 months	1.03	1.00	1.07	1.02	0.98	1.06
Social support	0.41***	0.28	0.59	0.48***	0.33	0.70
Antenatal depression				3.08***	2.00	4.74
History of depression				1.69	0.95	3.01

Data source: ELSPAC-CZ

N = 3005, *OR* odds ratio, *CI* confidence interval

**p < .01; ***p < .001 (two-tailed tests)

As the next step, an interaction between education and perceived discrimination was entered to assess whether the influence of perceived discrimination on postpartum depression varied among women with low vs. high levels of education (results not shown). The interaction term was significant (OR 2.24, *p* = .046), indicating that the effect of perceived discrimination is significantly stronger for women with low education compared to their highly educated counterparts. This result is consistent with the second hypothesis. To illuminate these differences, models were estimated separately by education level (Table 3). For women with low levels of education, the odds of postpartum depression increased more than two-fold with perceived discrimination (OR 2.43, *p* < .01) but for women with high education, the coefficient for perceived discrimination was not statistically significant.

**Table 3:** Estimates from general linear models of postpartum depression by education level

	Low Education			High Education		
	OR	95% CI		OR	95% CI	
Perceived discrimination	2.43**	1.29	4.56	1.06	0.61	1.84
Age at delivery, years	0.96	0.90	1.03	1.01	0.96	1.07
European ethnicity	0.41	0.09	1.79			
Has children	1.13	0.56	2.25	1.32	0.79	2.23
Married	1.56	0.74	3.32	2.92	0.88	9.69
Household size	1.16	0.98	1.37	1.04	0.86	1.27
Life events, prior 12 months	0.98	0.92	1.05	1.04	0.99	1.08
Social support	0.31***	0.17	0.56	0.65	0.39	1.07
History of depression	1.15	0.43	3.08	2.08*	1.01	4.29
Antenatal depression	3.77***	2.01	7.05	2.67**	1.44	4.97

Data source: ELSPAC-CZ. *OR* odds ratio, *CI* confidence interval. N = 1119 for low education. N = 1884 for high education. Ethnicity was dropped in the model for high education because only 2 participants in this group reported non-European ethnicity

*p < .05; **p < .01; ***p < .001 (two-tailed tests)

As expected, antenatal depression emerged as a strong predictor of postpartum depression in all models. In the fully controlled model using the whole sample (Table 2, Model 2), the odds of postpartum depression were 3.08 times higher (*p* < .001) for women with antenatal depression compared to those without antenatal depression. Antenatal depression was linked to higher odds of postpartum depression among women of both educational levels, with a stronger effect among women with low education (OR 3.77 vs. OR 2.67, p-values < 0.001). A history of major depression was related to 2.08 times higher odds of postpartum depression among highly educated women (*p* < .05). The likelihood of postpartum depression decreased with higher levels of social support (OR .31, *p* < .001) among women with low education.

## Discussion

Identifying factors that play a role in postpartum depression is an important component of the effort to prevent mental health problems among childbearing women. This study focuses on perceived discrimination as a risk factor. The results reveal that among women with low education, perceived discrimination reported during pregnancy is associated with an increased risk of depression assessed at six months post-delivery. Importantly, this effect is not explained by depression during and before pregnancy; even after accounting for the association between perceived discrimination and concurrently measured depression, perceived discrimination emerges as an independent predictor of future postpartum depression among lower SES women. These findings extend the literature on adverse outcomes of perceived discrimination, adding more specific evidence relevant to mental health of women who have recently given birth.

The results of this study are consistent with the bio-psycho-social model that suggests a causal role of perceived discrimination in a variety of negative health outcomes. Because of the longitudinal character of the data used here, we were able to assess the influence of perceived discrimination over time and provide a stronger test compared to earlier cross-sectional studies. Yet, potential confounding cannot be ruled out given the observational design. We made an effort to adjust for known socio-demographic covariates of depression as well as social support and life events as a measure of stress. Yet, some potentially relevant measures were not available, including self-esteem, locus of control, and coping styles. Future research must focus on these characteristics in the effort to understand factors that may help to protect mental health of women exposed to discrimination and socio-economic disadvantages.

Additionally, more detailed investigations are needed to better understand the mechanisms undergirding the key finding of this study; namely, that discrimination was linked to future depression among women with low education but not among their more educated counterparts. Stress process, which has been proposed as a link between discrimination and poorer health (Pascoe & Richman 2009), may work differently among socially advantaged vs. disadvantaged groups. Individuals with low SES often face multiple stressors in their lives. In addition to facing more discrimination, they are more commonly exposed to food insecurity, unemployment, financial difficulties, violence, physical and psychological abuse (especially for women), hazardous living conditions, and crime-ridden neighborhoods (Evans et al. 2011). It is possible that the harmful effects of each type of stressor are potentiated when other stressors are present as well. In addition, socio-economically disadvantaged individuals tend to have lower availability of stress buffers, such as social support (Campbell et al. 1986). Interestingly, social support in this research emerged as a significant protective factor against postpartum depression among poorly educated women but not among their well educated counterparts. These observations suggest that when social support is available for disadvantaged women, its benefits in terms of mental health surpass the benefits afforded to more advantaged women.

Among limitations of the sample are ethnic homogeneity and attrition patterns. The sample was remarkably homogeneous in terms of its ethnic composition, especially among participants with high education. According to The Czech Bureau of Statistics (2013), ethnic homogeneity is a defining characteristic of the Czech population in general. Given the specifics of the sample used here, the results should be further tested with more diverse populations outside of the Czech context and well as with samples specifically targeting minorities within the Czech Republic (as opposed to the general population).

In longitudinal designs, studying diverse populations is often complicated by non-random attrition. Similarly to other longitudinal studies, minority individuals in this study were more likely to drop out and the profile of drop-outs corresponds to prior studies as far as other demographic characteristics are concerned (Agosti et al. 1996; Fitzgerald et al. 1998). Drop-outs were also less likely to report antenatal depression and discrimination, making it important to take these attrition patterns into account when interpreting the results.

Finally, the limitations of the measure of perceived discrimination must be noted. First, perceived discrimination was assesed in mid-pregnancy and participants were asked to report experiences that happened to them during 12 months preceding the survey. This makes it impossible to determine whether the exposure to a discriminatory event occurred during pregnancy or during the pre-conception period. Second, pregnancy was not specified among potential reasons for discrimination. Yet, discrimination due to pregnancy is common, especially in the employment realm. Some employers prefer not to employ pregnant women because of potential losses in productivity and expenses linked to maternity leave. Because of these preferences, pregnant women may find it more difficult to obtain and keep a job. This can add to stress, especially for women who are facing economic difficulties. Since little is known about the effects of pregnancy-based discrimination on emotional well-being of victims, further investigation of this pregnancy-specific stressor is warranted.

The data limitations notwithstanding, this study, when considered together with the body of research documenting adverse effects of discrimination on health outcomes beyond postpartum depression, suggest that perceived discrimination represents a prominent health risk and should be considered as a candidate for screening during pregnancy. At this time, however, there is a need for the development of screening instruments focused on perceived discrimination that could be administered in the context of prenatal care. The potential utility of interventions for pregnant women who report discrimination and come from low socio-economic backgrounds must still be investigated. On the broader level of the society, policies that address discrimination should be a part of a comprehensive approach to women’s health. Despite anti-discriminatory policies, subtle forms of discrimination persist as a problem in many societies (Dovidio et al. 2008). Women, especially those with low SES, are at an increased risk of discrimination and depression across life-span (Schmitt et al. 2003; Van de Velde et al. 2010). This underscores the need for systemic initiatives to stem the damaging effects of discrimination on women’s health.

To conclude, this research identifies perceived discrimination as an independent risk factor for subsequent postpartum depression among women with low levels of education. Future research is needed to evaluate best approaches for addressing perceived discrimination along with other risks for maternal and child health, especially among disadvantaged populations. Such efforts could contribute to improving mental health and well-being for childbearing women.
